# Walking on inclines: how do desert ants monitor slope and step length

**DOI:** 10.1186/1742-9994-5-8

**Published:** 2008-06-02

**Authors:** Tobias Seidl, Rüdiger Wehner

**Affiliations:** 1 University of Zurich, Institute of Zoology and Center of Neuroscience, Winterthurerstrasse 190, 8057 Zurich, Switzerland; 2 European Space Agency, Advanced Concepts Team, Keplerlaan 1, 2201 AZ Noordwijk, The Netherlands

## Abstract

**Background:**

During long-distance foraging in almost featureless habitats desert ants of the genus *Cataglyphis *employ path-integrating mechanisms (vector navigation). This navigational strategy requires an egocentric monitoring of the foraging path by incrementally integrating direction, distance, and inclination of the path. Monitoring the latter two parameters involves idiothetic cues and hence is tightly coupled to the ant's locomotor behavior.

**Results:**

In a kinematic study of desert ant locomotion performed on differently inclined surfaces we aimed at pinpointing the relevant mechanisms of estimating step length and inclination. In a behavioral experiment with ants foraging on slippery surfaces we broke the otherwise tightly coupled relationship between stepping frequency and step length and examined the animals' ability to monitor distances covered even under those adverse conditions. We show that the ants' locomotor system is not influenced by inclined paths. After removing the effect of speed, slope had only marginal influence on kinematic parameters.

**Conclusion:**

From the obtained data we infer that the previously proposed monitoring of angles of the thorax-coxa joint is not involved in inclinometry. Due to the tiny variations in cycle period, we also argue that an efference copy of the central pattern generator coding the step length in its output frequency will most likely not suffice for estimating step length and complementing the pedometer. Finally we propose that sensing forces acting on the ant's legs could provide the desired neuronal correlate employed in monitoring inclination and step length.

## Background

Foraging desert ants, *Cataglyphis fortis *Forel 1902 [1], can navigate without using local external cues such as odor trails or visual landmarks. Instead they employ mechanisms of path integration [2], which actually is some kind of vector navigation [3]. It requires that the ant constantly updates its home vector, i.e. the location of the colony's nest defined by the direction and distance with respect to the animal's current position. In order to gather this information the ant must integrate each single increment of its path [4,5]. Therefore it must continually monitor the directions steered and distances covered in these directions. While compass information is gained by exploiting celestial cues (e.g. [6-8]; for reviews see: [1,9]), the mechanism of distance estimation has been a long standing problem. Flying insects rely on visual flow field cues (bees: [10-12]; wasps: [13]), but desert ants completely disregard lateral visual cues and incorporate ventral ones only to a degree of less than 10 per cent [14,15]. Energy expenditure as a measure of distance, as proposed by Heran and Wanke [16], can be ruled out as well, because distance measurement is not impaired in ants which on their inbound paths are loaded with food items [1]. With an elegant experiment Wittlinger and co-workers [17] proved a hypothesis first proposed by Pieron [18] that some kind of step counter is responsible for distance estimation. However, as step length varies with speed of locomotion [19], a mere step counter will not work [20]. In order to calculate a distance correctly, both step number and corresponding step length need to be integrated. So far it is unclear how step length is monitored. According to [20,21] step length and step frequency are tightly coupled within an individual ant. Hence, the output frequency of a central pattern generator (e.g. [22]) might already contain the information required for distance estimation as has been mentioned in a previous study [20].

But vector navigation does not only take place on level ground: the ant's path integration system is able to compensate for vertically corrugated paths. Wohlgemuth and co-workers [23,24] showed that ants were able to correctly calculate the ground distance between feeder and nest even if the ants had performed their previous outbound runs on a corrugated surface and hence had covered much longer paths.

Consequently, the animals must have a representation of the slope of their outbound paths, and based on this representation they must have computed the corresponding ground projection (base-line distance) of their three-dimensional path. The mechanisms responsible for slope detection most likely involve idiothetic cues. For obvious reasons, energy expenditure will be a misleading cue as running up and down a corrugated path will require more energy than to take the short and level projection path. Visual input by skylight cues which change their position in the visual fields of uphill and downhill running ants was also excluded by Wohlgemuth and co-workers [24]. In insects, slope (via gravity) can be monitored by idiothetic cues either by hair field sensors between body parts [25] or by campaniform sensilla within the cuticle of the legs of running insects [26]. As Wittlinger and co-workers [27] had deactivated hair sensilla on the ant's body without causing any impairment on the ants' ability to integrate their paths on corrugated sheets, we now focus on the legs and their movement parameters as a source of information potentially used by the ants, i.e. in measuring the inclination of the surface on which they walk.

In summary, idiothetic cues are the most plausible mechanisms for being involved in measuring both step length and slope during three-dimensional path integration. In general, we consider three types of parameters and their possible use in accomplishing this task: (i) Does the angular working area of the legs vary with inclination, including a resulting change in step length? (ii) Do temporal patterns such as swing phase and stance phase depend on the inclination of the walking floor? Finally, (iii) do forces acting along the legs during the stance phase of the stepping cycle change with inclines and with step length, and hence would they be able to serve as a cue for three-dimensional path integration? We designed two types of experiments addressing these questions: First, we studied the kinematics of leg movements of ants of two species running on differently inclined surfaces. This study should show whether the first two groups of parameters mentioned above, i.e. geometric and temporal variations of leg movements, would play a role. Our desert-living model animal was compared with wood ants that are confronted with a habitat that allows for entirely visual navigation and hence will allow us to generalize our findings. Second, we performed a behavioral experiment with ants foraging on slippery substrates and therefore being prevented from producing high mechanical forces along their legs. This study should show whether force-monitoring is a mechanism potentially employed in path integration.

## Results

### Locomotion on inclines

**Figure 1:**
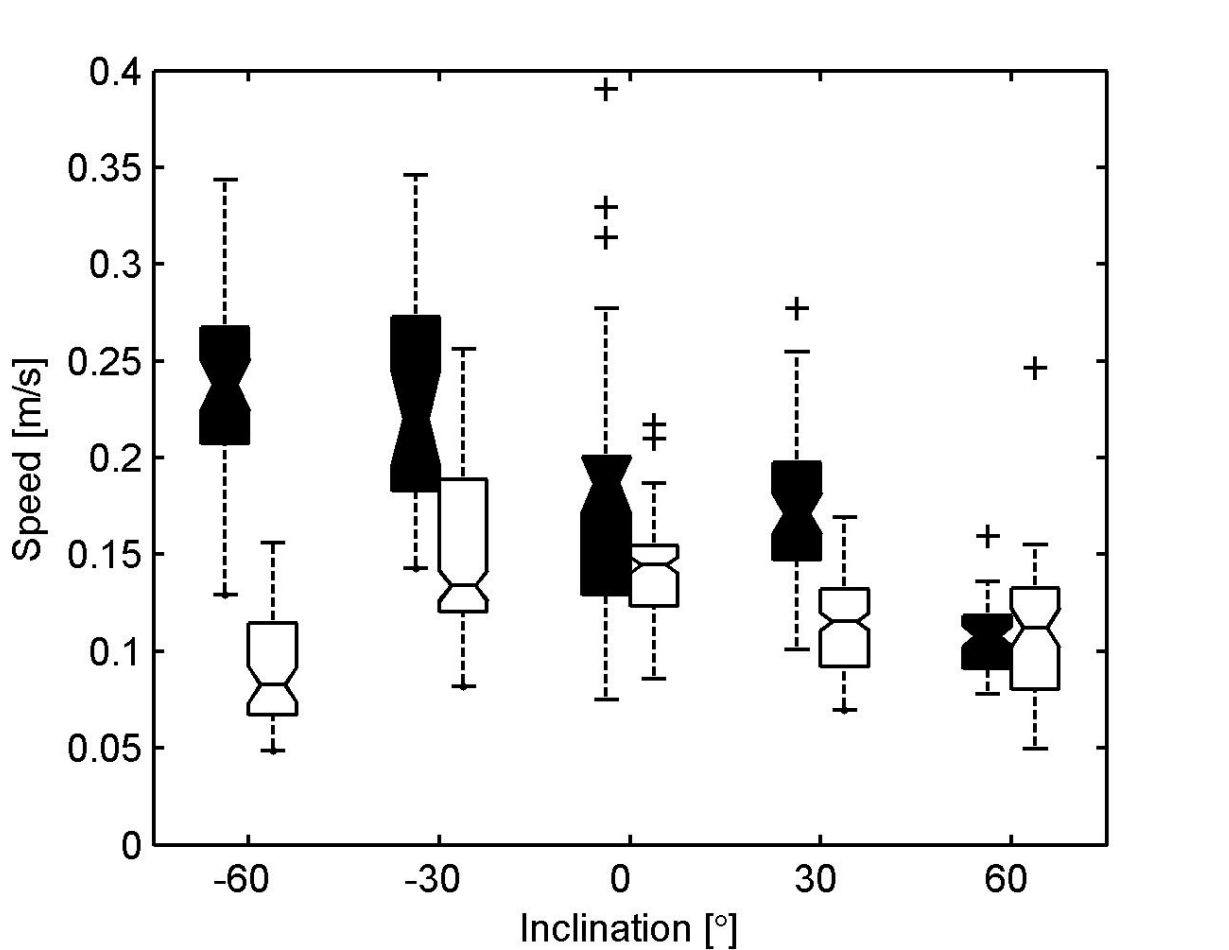
Speed of locomotion of *C. fortis *(filled boxes) and *F. pratensis *(empty boxes) at different inclinations. Boxplots with 25%, 50% and 75% percentiles; whiskers extend to max. 1.5 times the inter-quartile range. Notches indicate significant difference in pairwise comparisons.

For the kinematic analysis we recorded, from a dorsal perspective, 263 sequences of ants running on inclines. Each sequence contained one to twelve steps analyzed (mean: 3 steps). This resulted in a total of 876 steps. Separated by species and inclination the numbers are: inclination of -60° : 47 and 67 steps (*C. fortis *and *F. pratensis*, respectively), -30° : 33 and 181 steps, 0° : 54 and 127 steps, 30° : 54 and 186 steps,60° : 64 and 63 steps. Negative inclinations denote downhill paths. The examined ants ran at speeds between 0.05 and 0.4 m/s. The effect of slope on speed of locomotion differed between the two species (Figure 1). Concerning the median values, desert ants maintained high velocities on downhill paths and run slowest on steep uphill paths. Wood ants in contrast maintained fairly constant running speeds at all conditions except steep downhill paths. While transfer from kinematic to potential energy may explain the desert ants' values, the wood ants' set indicates some kind of safety behavior in an extreme situation. Concerning maximum speeds desert ants ran fastest on level ground (0.4 m/s or about 40 body lengths per second), while wood ants accelerated most on modest downhill paths (0.25 m/s, -30°). In consequence, ants of either species ran at the same speed of 0.1 m/s on steep uphill paths (60°), but they differed extremely on steep downhill slopes (-60°): 0.07 m/s (wood ants) and 0.23 m/s (desert ants). Due to this divergence we removed the effect of speed from the data for all further analyses.

The major aim of this study was to identify effects of the inclination of the walking floor on the kinematics of the ants' locomotor behavior. In order to separate any potential inclination-specific effect from a species-or habitat-specific effect, we compared our study animal *Cataglyphis fortis *with wood ants, *Formica pratensis*. While the desert ants are bound to rely on precise path integration mechanisms, wood ants inhabit a rather structured environment and, hence, may – hypothetically – employ less accurate egocentric navigation and rely instead more on external visual cues (see also: [28,29]). Due to experimental peculiarities we included both head width and speed of locomotion into the analysis. In consequence, the statistical tool is a multivariate analysis with four input variables according to the formula displayed in the Material and Methods section. The input variables are speed of locomotion, species (considering allometric morphological variations between species), head width (considering within-species size effects) and slope. In the following we developed several hypotheses on monitoring step length and slope and tested these hypotheses by determining the influence of the four input variables on the specific output variable addressed by the particular hypotheses. The model parameters of the input variable denote the degree of their influence on the output variable. Together with the corresponding p-values they are listed in Tables 1 and 2. The results described in the following are presented as answers to six questions (hypotheses) on how ants might integrate paths while walking on inclines. As mentioned above, ants are able to compute ground distance covered on hilly paths, and hence must be able to monitor the inclination of the floor on which they walk.

**Table 1:** Influence of the four input parameters on geometric kinematic parameters.

**Parameter**	**Leg**	*α ***Speed [m/s] **mean ± s.d.	*β ***Species [Cf, Fp] **mean ± s.d.	*γ ***Head width [mm] **mean ± s.d.	*δ ***Inclination [°]**mean ± s.d.	*ε ***Intercept **mean ± s.d.	**log-likelihood**
	
**Step length^**(2) **^[mm]**	f^(3)^	29.8 ± 1.4^****(1)^	-0.51 ± 0.22*	0.55 ± 0.34^*n*.*s*.^	-0.005 ± 0.003^*n*.*s*.(2)^	3.96 ± 0.77****	-1322
	m^(3)^	28.7 ± 1.2****	-0.50 ± 0.20*	0.85 ± 0.30**	-0.004 ± 0.003^*n*.*s*.(2)^	3.53 ± 0.68****	-1467
	h^(3)^	29.7 ± 1.2****	-0.33 ± 0.20^*t*.^	0.72 ± 0.30*	-0.006 ± 0.003^*(2)^	3.55 ± 0.69****	-1424
**Step length [mm]**	f	30.1 ± 1.4****	-0.46 ± 0.22*	0.57 ± 0.34^*t*.^	-0.0005 ± 0.0019^*n*.*s*.^	3.67 ± 0.76****	-1324
	m	29 ± 1.3****	-0.45 ± 0.2*	0.87 ± 0.3**	-0.0002 ± 0.0017^*n*.*s*.^	3.29 ± 0.68****	-1469
	h	30.1 ± 1.3****	-0.27 ± 0.2^*n*.*s*.^	0.74 ± 0.31*	-0.0007 ± 0.0017^*n*.*s*.^	3.21 ± 0.69****	-1427
**AEP [mm] (cranial)**	f	3.99 ± 1.07***	-0.74 ± 0.16****	0.58 ± 0.25*	0 ± 0.0014^*n*.*s*.^	4.23 ± 0.56****	-1459
	m	3.13 ± 1.2**	0.38 ± 0.18*	0.47 ± 0.27^*t*.^	-0.0002 ± 0.0015^*n*.*s*.^	0.65 ± 0.61^*n*.*s*.^	-1653
	h	0.1 ± 1.09^*n*.*s*.^	1.78 ± 0.17****	-0.33 ± 0.25^*n*.*s*.^	-0.0019 ± 0.0014^*n*.*s*.^	-4.05 ± 0.57****	-1446
**AEP [mm] (distal)**	f	-0.6 ± 0.81^*n*.*s*.^	-1.18 ± 0.12****	-0.07 ± 0.18^*n*.*s*.^	-0.0022 ± 0.001*	2.9 ± 0.41****	-1309
	m	3.02 ± 0.83***	-2.02 ± 0.13****	0.16 ± 0.2^*n*.*s*.^	0.0001 ± 0.0011^*n*.*s*.^	5.39 ± 0.45****	-902
	h	0.86 ± 0.8^*n*.*s*.^	-1.19 ± 0.12****	0.3 ± 0.19^*n*.*s*.^	0.0011 ± 0.0011^*n*.*s*.^	3.39 ± 0.42****	-1115
**PEP [mm] (cranial)**	f	0.68 ± 0.71^*n*.*s*.^	-0.81 ± 0.11****	0.46 ± 0.17**	0.0014 ± 0.0009^*n*.*s*.^	1.42 ± 0.37****	-950
	m	-1.83 ± 0.84*	0.63 ± 0.13****	0.2 ± 0.19^*n*.*s*.^	0.0001 ± 0.0011^*n*.*s*.^	-2.47 ± 0.43****	-1311
	h	-0.4 ± 1.03^*n*.*s*.^	2.01 ± 0.16****	-0.59 ± 0.25*	-0.0008 ± 0.0014^*n*.*s*.^	-7.24 ± 0.56****	-1104
**PEP [mm] (distal)**	f	-1.44 ± 0.74^*t*.^	-1.4 ± 0.11****	-0.06 ± 0.17^*n*.*s*.^	-0.0031 ± 0.001**	3.1 ± 0.38****	-1182
	m	4.09 ± 0.82****	-1.68 ± 0.13****	0.23 ± 0.2^*n*.*s*.^	0.0009 ± 0.0011^*n*.*s*.^	4.86 ± 0.44****	-961
	h	-0.29 ± 0.86^*n*.*s*.^	-1.45 ± 0.13****	0.28 ± 0.2^*n*.*s*.^	0.0005 ± 0.0011^*n*.*s*.^	3.71 ± 0.45****	-1289

^(1)^) Levels of significance: **** p < 0.0001; *** p < 0.001; ** p < 0.01; * p < 0.05; t. p < 0.1; n.s. p ≥ 0.1.

^(2)^) absolute values.

^(3)^) f: front, m: middle, h: hind.

Each line represents one statistic model. The output parameter is given in via the two left columns – e.g. step length of the front leg. The values given in the columns of the four input parameters speed, species, head width, and inclination represent the strength of their influence on the variation of the output parameter. The statistic significance reflects upon the validity of the fit. (Input parameters: 258 degrees of freedom, intercept: 613 degrees of freedom)

**Table 2:** Influence of the four input parameters on temporal kinematic parameters. (Refer to legend of Table 1.)

**Parameter**	**Leg**	*α ***Speed [m/s] **mean ± s.d.	*β ***Species [Cf, Fp] **mean ± s.d.	*γ ***Head width [mm] **mean ± s.d.	*δ ***Inclination [°] **mean ± s.d.	*ε ***Intercept **mean ± s.d.	**log-likelihood**
	
**Cycle period [s]**	f^(3)^	-0.2 ± 0.01^****(1)^	-0.004 ± 0.002^*t*.^	0.002 ± 0.003^*n*.*s*.^	-0.00005 ± 0.00002**	0.09 ± 0.01****	2638
	m^(3)^	-0.22 ± 0.01****	-0.005 ± 0.002*	0.003 ± 0.003^*n*.*s*.^	-0.00007 ± 0.00002***	0.92 ± 0.01****	2585
	h^(3)^	-0.23 ± 0.01****	-0.004 ± 0.002^*t*.^	0.003 ± 0.003^*n*.*s*.^	-0.00006 ± 0.00002***	0.09 ± 0.01****	2569
**Frequency**^(2) ^**[Hz]**	f	54.9 ± 4.6****	0.9 ± 0.71^*n*.*s*.^	-0.29 ± 1.07^*n*.*s*.^	0.013 ± 0.006*	9.3 ± 2.4***	-2590
	m	58 ± 2.9****	0.4 ± 0.43^*n*.*s*.^	-1.21 ± 0.65^*t*.^	0.006 ± 0.004^*n*.*s*.^	10.8 ± 1.5****	-2365
	h	56.9 ± 3.4****	0.16 ± 0.52^*n*.*s*.^	-1.22 ± 0.78^*n*.*s*.^	0.006 ± 0.004^*n*.*s*.^	11 ± 1.8****	-2446
**Swing phase [s]**	f	-0.051 ± 0.007****	-0.0024 ± 0.0011*	0.0027 ± 0.0016^*t*.^	-0.00002 ± 0.00001*	0.033 ± 0.004****	3162
	m	-0.043 ± 0.007****	-0.0003 ± 0.0011^*n*.*s*.^	0.0038 ± 0.0016*	-0.00003 ± 0.00001**	0.024 ± 0.004****	3235
	h	-0.051 ± 0.008****	-0.0012 ± 0.0012^*n*.*s*.^	0.0033 ± 0.0018^*t*.^	-0.00002 ± 0.00001^*t*.^	0.032 ± 0.004****	3015
**Stance phase [s]**	f	-0.15 ± 0.01****	-0.0009 ± 0.0015^*n*.*s*.^	-0.0003 ± 0.0022^*n*.*s*.^	-0.00004 ± 0.00001**	0.056 ± 0.005****	2691
	m	-0.18 ± 0.01****	-0.004 ± 0.0016*	0.0001 ± 0.0024^*n*.*s*.^	-0.00003 ± 0.00001*	0.067 ± 0.005****	2607
	h	-0.18 ± 0.01****	-0.0028 ± 0.0017^*t*.^	0.0002 ± 0.0026^*n*.*s*.^	-0.00004 ± 0.00001**	0.061 ± 0.006****	2683
**Duty Factor [-]**	f	-0.73 ± 0.09****	0.03 ± 0.013*	-0.015 ± 0.02^*n*.*s*.^	-0.00008 ± 0.00011^*n*.*s*.^	0.63 ± 0.05****	684
	m	-0.75 ± 0.1****	-0.012 ± 0.015^*n*.*s*.^	-0.035 ± 0.022^*n*.*s*.^	0.00006 ± 0.00013^*n*.*s*.^	0.77 ± 0.05****	672
	h	-0.95 ± 0.1****	0.003 ± 0.015^*n*.*s*.^	-0.012 ± 0.023^*n*.*s*.^	-0.00012 ± 0.00013^*n*.*s*.^	0.68 ± 0.05****	658
**Within tripod phase shift [-]**	R1	3.78 ± 0.66****	0.39 ± 0.1***	0.16 ± 0.15^*n*.*s*.^	0.0011 ± 0.0009^*n*.*s*.^	1.9 ± 0.3****	-1093
	R3	-0.81 ± 0.66^*n*.*s*.^	-0.07 ± 0.1^*n*.*s*.^	-0.03 ± 0.15^*n*.*s*.^	0.0001 ± 0.0009^*n*.*s*.^	3.5 ± 0.3****	-1072
**Alternate tripod phase shift [-]**	L1	1.98 ± 0.45****	0.18 ± 0.07**	0.05 ± 0.1^*n*.*s*.^	-0.0001 ± 0.0006^*n*.*s*.^	-0.4 ± 0.2^*t*.^	-686
	R2	0.22 ± 0.62^*n*.*s*.^	0.08 ± 0.09^*n*.*s*.^	0.05 ± 0.14^*n*.*s*.^	0.0005 ± 0.0008^*n*.*s*.^	2.7 ± 0.3****	-1084
	L3	-0.96 ± 0.42*	-0.22 ± 0.06***	-0.11 ± 0.1^*n*.*s*.^	-0.0007 ± 0.0005^*n*.*s*.^	0.8 ± 0.2***	-712

^(1)^) Levels of significance: **** p < 0.0001; *** p < 0.001; ** p < 0.01; * p < 0.05; t. p < 0.1; n.s. p ≥ 0.1.

^(2)^) Frequency is the inverse of cycle period; the latter value was actually the one analyzed directly.

^(3)^) f: front, m: middle, h: hind.

### Geometric parameters

**Figure 2:**
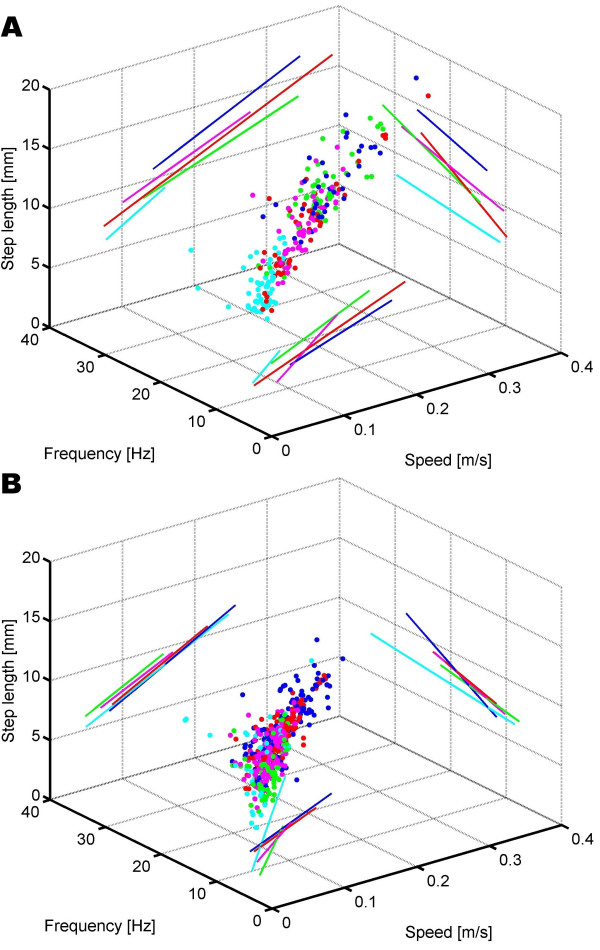
Relationship between speed, step length, and stepping frequency at different inclinations in *C. fortis *(a) and *F. pratensis *(b). Dots: values plotted in 3D space; lines: projected linear fits of data on two parameters; green: -60°, blue: -30° ; red: 0° ; magenta: 30°, cyan: 60°.

Our first and quite provocative hypothesis on the mechanisms of path integration on inclined paths states: "*With increasing slope, path-integrating ants increase their step length in such a way that for a given base-line distance covered the number of steps remains constant irrespective of the inclination of the floor on which the ants have actually walked*." In this first analysis we did not differentiate between uphill and downhill runs and hence used only absolute values for the inclination of the plane in which the ants walked. The desert ants made steps of 10.1 ± 2.5 mm (all values: mean ± s.d.), while the wood ants had step lengths of 7.9 ± 1.3 mm (Figure 2). As desert ants make longer steps than wood ants do the species effect has a negative value: *β*≈-0.4 mm (compare Material and Methods section and Table 1). In addition there is also an effect of individual body size: larger individuals made longer steps (*γ*≈0.8 mm step length per 1 mm head width), i.e., an increase in head width of 1 mm is correlated with an increase in step length of 0.8 mm. The major effect on step length, however, is speed of locomotion (*α*≈29 mm per m/s), i.e. an ant accelerating for 0.1 m/s elongates its steps by 2.9 mm. Slope, however, has a significant influence on step length only in the hind leg (*δ *= -0.0063 ± 0.003 mm per degree inclination). In *Cataglyphis*, an increase of inclination of, e.g., 30° would result in a shortening of step length by 0.2 mm. The influence of inclination on step length can also be read of Figure 2: The inclination-wise linear fits of step length versus speed do not differ considerably. Our first hypothesis – steps get longer as inclination increases – is herewith rejected. Even if step length is modeled by differentiating between uphill and downhill runs, as done in all following analyses, the *δ*-values do not become significant; all other model-parameters remain fairly the same. Walking on an inclined surface requires more force, because lift of the whole body has to be generated. While running on level ground, the vector of gravity is oriented vertically to the surface and so is normal to the horizontally produced thrust. On inclines, however, there is always a component of the gravitational force acting along the direction of locomotion, pulling the ant anterior or posterior. Hence, there is additional acceleration acting on the body which has to be integrated while generating the desired anterior thrust.

**Figure 3:**
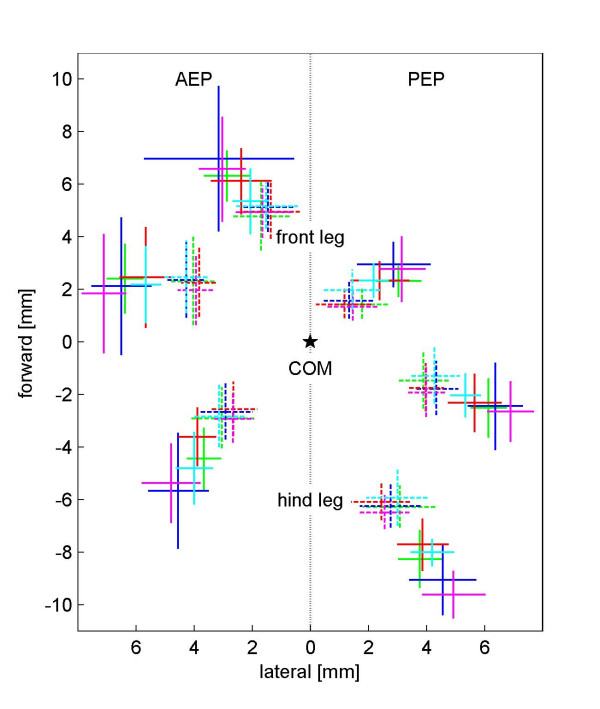
Footfall geometry of *C. fortis *(solid lines) and *F. pratensis *(dashed lines) during touch down (AEP) and lift off (PEP) at different inclinations. The intersection of two lines denotes the mean value, while the ends of the lines denote the standard deviation. Star: center of mass (COM); green: -60°, blue: -30° ; red: 0° ; magenta: 30°, cyan: 60°. Spatial resolution: 0.1 mm.

**Figure 4:**
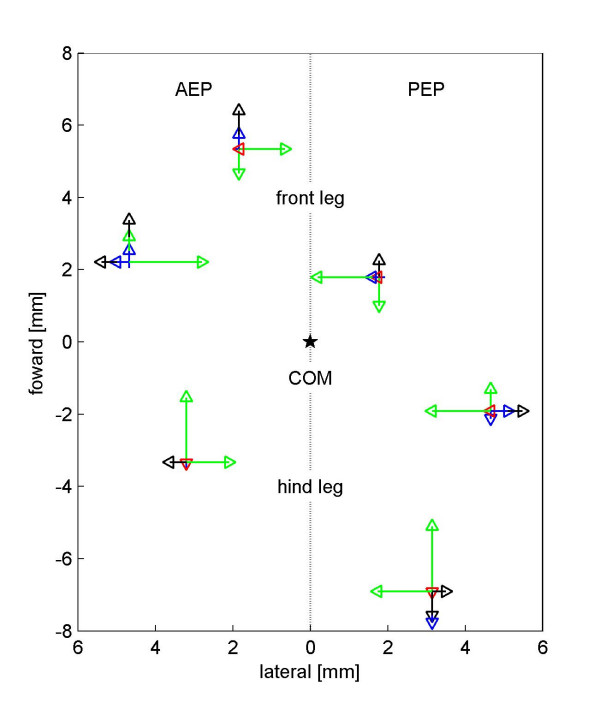
Influence of the input parameters on footfall geometry during the touch down (AEP) and the lift off (PEP) phase at different inclinations. The influences of speed (blue, per 0.1 m/s acceleration), species (green, from *C. fortis *to *F. pratensis*), head width (black, per 1 mm increase in head width), and inclination (red, per an increase of 10° in inclination) are plotted on the mean value of all analyzed runs. Arrows are attached to each other, i.e., if pointing in the same direction, one arrow has its base at the tip of the previous one.

In order to maintain static stability, desert ants could adapt their locomotive behavior by moving the stance phase in direction of the gravity vector, similar to a person tilting its body toward a strong wind. These considerations lead to our second hypothesis on path integration in the third dimension: "*On uphill paths the footfall positions are moved in a posterior direction (caudally), and on downhill paths they are moved in an anterior direction (cranially)*." Such a behavior would allow the ant to keep its center of mass (COM) longer in the static safe position above the supporting tripod-triangle. After a swing phase, the tarsi reach their most anterior point just when they touch down. At this phase of the stepping cycle – the anterior extreme position (AEP) – most of the variation that can be observed is due to the species effect: In relation to desert ants, wood ants position their front legs posteriorly (*β *= -0.74 ± 0.16 mm) and proximally (*β *= -1.18 ± 0.12 mm), their middle legs are placed slightly anteriorly (*β *= 0.38 ± 0.18 mm) and proximally (*β *= -2.02 ± 0.13 mm), while their hind legs touch the ground in both an anteriorly (*β *= 1.78 ± 0.17 mm) and proximally translated position (*β *= -1.19 ± 0.12 mm). These species effects can be observed with similar values at the end of the stance phase, the posterior extreme position (PEP): Again, if compared to the desert ants, the front legs of the wood ants move posteriorly and proximally (*β *= -0.81 ± 0.11 and -1.4 ± 0.11 mm), the middle legs slightly anteriorly and proximally (*β *= 0.63 ± 0.13 and -1.68 ± 0.13 mm), and the hind legs anteriorly and proximally (*β *= 2.01 ± 0.16 and -1.45 ± 0.13 mm). In four cases, the individual body sizes of conspecific ants have statistically significant influences (or in tendency, i.e. 0.05<p < 0.1): The front leg is moved anterior in both AEP and PEP (*γ *= 0.58 ± 0.25 and 0.46 ± 0.17 mm per mm head width). The middle leg is, in tendency, positioned anteriorly (*γ *= 0.47 ± 0.27 mm per mm head width) in AEP; the hind leg is positioned posteriorly (*γ *= -0.59 ± 0.25 mm per mm head width) in PEP. During touch down, fast running ants place their front and middle legs anteriorly (*α *= 3.99 ± 1.07 and 3.13 ± 1.2 mm per m/s) and their middle legs distally (*α *= 3.02 ± 0.83 mm per m/s). At the end of the stance phase (PEP), high speed of locomotion induces ants to move their middle legs further posteriorly (*α *= -1.83 ± 0.84 mm per m/s) and distally (*α *= 4.09 ± 0.82 mm per m/s), as well as their front legs proximally (*α *= -1.44 ± 0.74 mm per m/s). Slope, however, has a significant effect on AEP and PEP geometry only on the front legs: in both cases the front tarsus is positioned proximally (*δ *= -0.002 ± 0.001 mm and -0.003 ± 0.001 mm per degree inclination). A graphical display of the data is shown in Figure 3; the different effects on the positioning of the tarsi during AEP and PEP are shown in Figure 4. There is no visible anterior or posterior shift of AEP or PEP which could indicate an inclination-dependent response. Hence, our second hypothesis can clearly be rejected. Geometric factors such as step length or the standing triangle of the tripod do not vary with inclines. In other words, monitoring the positions of the legs does not provide the ants with information on the inclination of the substrate on which they walk.

### Temporal parameters

Legged locomotion is performed by a cyclic posterior and anterior movement of the animal's legs. During the posterior movement of the legs, i.e., during the stance phase, thrust is generated and the animal is pushed in an anterior direction. During the swing phase the legs are lifted up into the air and brought anterior in order to initiate a new stance phase. During this swing phase, the thin and light legs are only lifted against gravity and then swung horizontally. If the animal runs on an inclined path, gravity acts on the animal's body pulling it toward the surface of locomotion as well as downhill along the surface. The swinging legs will have to work against (or with) an additional acceleration pulling the legs posteriorly or anteriorly. This situation does not occur during level running. The mechanically otherwise uncoupled leg could function as a detector for external accelerations, similar to the widely spread antennae on the ant's head or even to the acceleration sensitive and actively moved halteres of dipteran and strepsipteran insects. Hence our third hypothesis on monitoring gravity for path integration reads: "*On uphill paths, the swing phase of the stepping cycle will be longer than on level ground, as additional lift needs to be generated. Accordingly, swing phases will be shorter on downhill paths*."

Swing phases in desert ants amount to 0.027 ± 0.004s and in wood ants to 0.028 ± 0.006s. The swing phases of the front legs differ between the two species significantly (*β *= -0.0024 ± 0.0011s for wood ants). The swing phase is significantly reduced by speed of locomotion (*α*≈-0.048s per m/s, Table 2), and is reduced in tendency by body size (*γ*≈-0.0032s per mm head width). Additionally there is a significant influence of inclination on swing phase (*δ*≈-0.00002s per degree inclination). However, the influence is rather small (e.g.0.0006s shortening at a slope increase of 30°) and below the temporal resolution of our measuring device (250 frames per second, or 0.004 seconds), so that the effect of gravity on the ant's swinging leg can be neglected. A graphical representation of these findings is shown in Figure 2: The inclination-wise linear fits of stepping frequency versus speed of locomotion do not differ in either intercept or slope.

But what happens to the animal's entire body, which obviously weighs a manifold (≈20 times) of a single leg? In this case, much more energy is required to produce lift and thus generate potential energy. On the other hand this potential energy can be released on downhill paths and can enable the animal to make use of this external source of energy. Based on this consideration, our fourth hypothesis reads: "*On uphill paths the stance phase will require more time, as additional lift for the entire body needs to be generated. To the contrary, a shortening of the stance phase is to be expected on downhill paths*."

In *Cataglyphis *ants stance phases last for 0.032 ± 0.011s, while in *Formica *ants the duration of the stance phase is 0.039 ± 0.012s. The major influence on stance phase is speed of locomotion (*α*≈-0.017s per m/s), and there is no significant effect of body size. Compared between species, wood ants display a shorter stance phase in the middle leg (*β *= -0.004 ± 0.002s) than desert ants. Inclination has a significant effect on the stance phase in all three legs (*δ*≈-0.00004s per degree inclination). Increasing slope by, e.g., +30° leads to a shortening of the stance phase of 0.0012s, again below the temporal resolution of our setup. The observed value is not in agreement with our prediction, which states that the stance phase should be elongated on uphill walks (positive slopes). Obviously, there is no direct effect of gravity on stance phase. Both major phases of the step cycle show a statistically significant effect toward inclination. In both cases the effect is marginal and below the temporal resolution of our setup. However, since they have an identical sign, the complete cycle exhibits a statistically significant effect as well: A shortening of the step cycle of 0.00006s per degree inclination leads to a mean shortening of the step cycle between two inclinations measured of 0.0018s, again not exceeding the temporal resolution of our recordings of 0.004 seconds between two consecutive frames. Only when comparing the most extreme situations (-60° and +60°) the variation of step cycle period adds up to -0.006s to -0.008s and hence exceeds the threshold of our acquisition system.

Walking uphill does not only require working against a downward acceleration. The additionally produced force also needs to be transmitted to the ground, i.e., the animal needs to establish a certain grip between tarsi and substrate. In both cases, the production of maximum force and grip is a direct function of the number of legs that at any time are in contact with the ground. Would an ant by trying to avoid uncontrolled and possibly fatal translations adapt its walking behavior on an inclined surface by taking extra measures of security? Our fifth hypothesis thus claims that "*ants adapt their leg coordination in order to produce more thrust and better surface attachment during uphill locomotion*". One way of 'keeping more feet on the ground' is changing one's gait: Ants running on level ground employ a alternating tripod gait. But insects can also use a tetrapod gait (e.g. [30]), which allows for more simultaneous foot-ground contacts and is therefore preferably used in climbing insects. The phase relationships of the legs were determined relative to the stepping cycle of the left middle leg (L2; phase = 0: touch down of L2 initiating the stepping cycle analyzed, phase = 1: subsequent touch down of L2 initiating the next stepping cycle). The mean phase shifts of the within-tripod legs are R1: 0.01 ± 0.09 and 0.02 ± 0.08 (*C. fortis *and *F. pratensis*, respectively); R3: 0.06 ± 0.11 and 0.04 ± 0.08. The legs of the alternate tripod show the following phase values (again compared to L2): L1: 0.47 ± 0.15 and 0.49 ± 0.13; R2: 0.46 ± 0.16 and 0.46 ± 0.12; L3: 0.53 ± 0.15; and 0.52 ± 0.12. The phase of the front legs is positively influenced by speed (R1: *α *= 3.78 ± 0.66; L1: *α *= 1.98 ± 0.45 per m/s), i.e., with higher speeds the touch down of the front legs is delayed. The touch down of the ipsilateral hind leg (L3), however, happens earlier with higher speeds: *α *= -0.96 ± 0.42. The same tendency, but with values of an order of magnitude lower, can be observed between species: The wood ants' front legs touch the ground later (*α *= 0.39 ± 0.10 and 0.18 ± 0.07), while the ipsilateral hind leg (L3) touches the ground earlier (*α *= -0.22 ± 0.06). There is no influence of individual body size or of slope on phase relationships. In conclusion, the ants remained faithful to their tripod gait throughout all steps analyzed but demonstrated a slight adaptation toward speed. There is no gait change at inclines of up to 60°.

Static stability can also be enhanced without gait transitions: The duty factor describes the fraction of the stance phase in relation to the whole cycle period. The higher the (gait independent) duty factor, the longer a foot has contact with the ground. The steps analyzed reveal a duty factor of 0.52 ± 0.08 for desert ants and 0.58 ± 0.06 for wood ants. Speed is the major influence on duty factor (*α*≈-0.8 per m/s), i.e., at higher speeds the relative ground contact time diminishes. In wood ants, only the front leg shows a significantly higher duty factor than in desert ants (*β *= 0.03 ± 0.01). Individual body size and inclination have no influence on duty factor. In conclusion, safety parameters such as gait and duty factor are not influenced by slope. The locomotive system of both species of ants is very robust toward inclinations. The only parameter that varies greatly with inclination is speed of locomotion (Figure 1). The fitted output parameters depend either on speed or on body size. Species effects occur mainly with respect to geometric parameters. Significant influences of slope occur mainly in temporal parameters but the effects are marginal.

Our results clearly show that the temporal parameters of ant locomotion are fairly robust against variations in slope. In addition, there is hardly any difference between the two species, and size effects are small. Hence, it is no surprise that the frequency of the whole stepping cycle behaves accordingly. In all three legs, stepping frequency correlates with speed (*α*≈56 Hz per m/s); in other words, speed is gained through a shortening of cycle period (*α*≈-0.22s per m/s). A significant species effect is found only in stepping period, but remains at a comparatively low value (*β*≈-0.004s in wood ants). Individual size influences the stepping frequency of the middle leg (*δ *= -1.21 ± 0.65 Hz per mm). The influence of slope is significant on stepping period (*δ*≈-0.00006s per degree), and in the front leg it is significant on stepping frequency (*δ *= 0.013 ± 0.006 Hz per degree). In both cases, the *δ *-values are close to zero and hence can be considered irrelevant. Even though stepping frequency and stepping period are reciprocals of each other, the goodness of fit-criteria, the log-likelihoods, differ considerably: The linear fits of the frequency and the stepping period produce log-likelihoods of ≈-2500, and ≈+2500, respectively. In a direct comparison with identical degrees of freedom (which is the case in our analysis), the higher value denotes a better fit. Hence, the stepping period allows for better linear fits than the stepping frequency. The slightly curve-like distribution of stepping frequency is also apparent in Figure 2.

#### Path integration on a slippery surface

**Figure 5:**
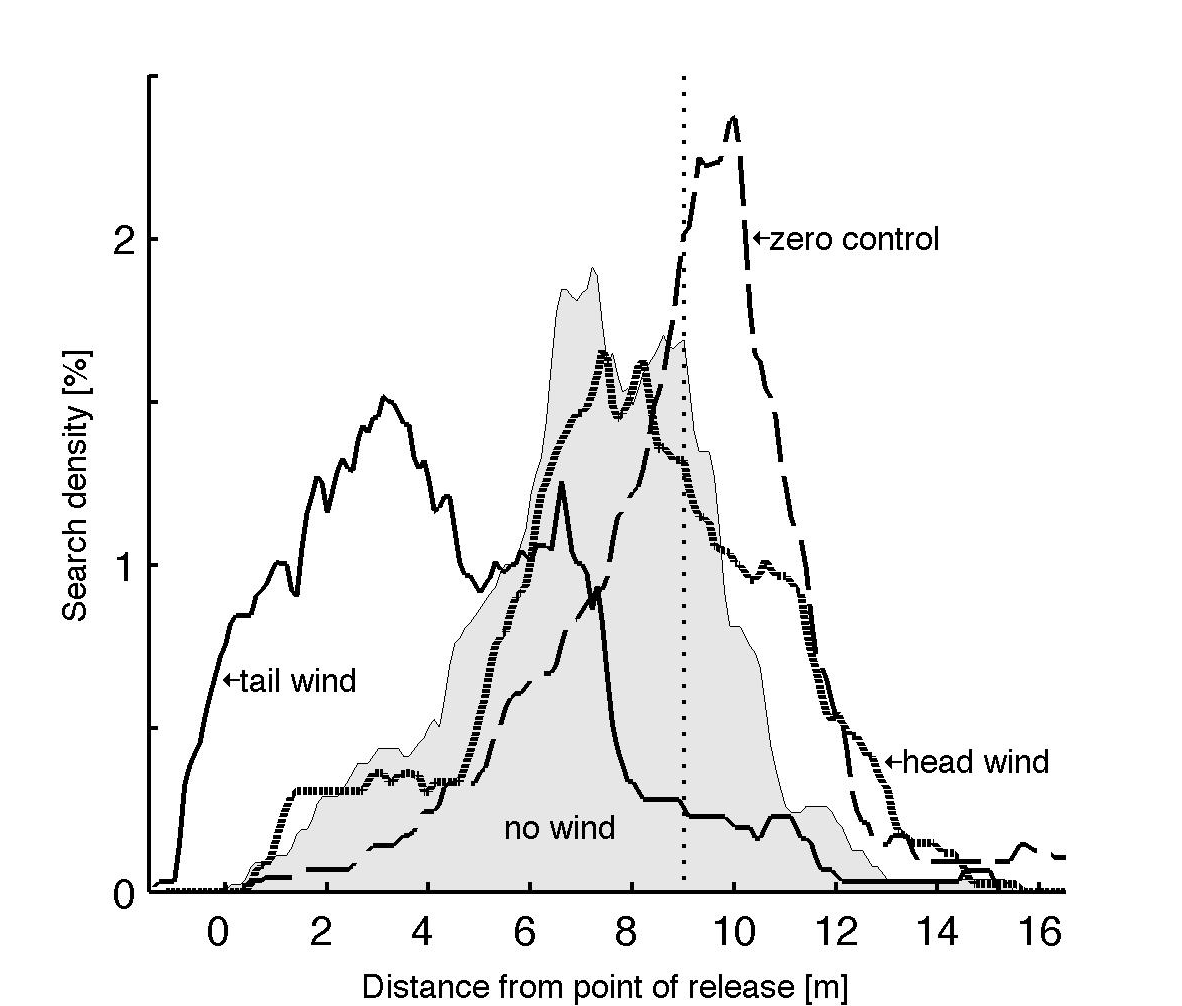
Density distributions of the ants' nest search behavior after the ants had performed their outbound runs on slippery ground (except for the zero control ants) under different wind conditions (tail wind: n = 13, no wind: n = 15, head wind: n = 18, zero control: n = 20).

As documented by the results of our kinematic study and together with the work of [27,31,32] we conclude that ants do almost not adapt the kinematics of their walking behavior to the inclination of the surface on which they walk. In conclusion, as external (gravitational) forces act on the running ant, the production of force output has to vary, if a constant behavior is to be maintained on all inclinations. This seems to be the case according to the data presented above. The question remains, how path integration on inclines may be performed, assuming that idiothetic cues are responsible for inclinometry. If force production at a given speed is a function of inclination, force sensitive receptors could play an important role in path integration. Should one be able to break the relationship between net shear force and step length experimentally, the ants should encounter difficulties in correctly estimating distances covered. Hence our sixth and final hypothesis is the following: "*Desert ants running on a slippery surface will experience increased tarsal slipping and hence restricted transmission of shear force to the ground. This effect eventually increased by external forces such as wind will cause the ants to misjudge their distances covered*." As running on a slippery surface is a rather challenging task, for a human as well as an ant, we performed this behavioral study only on level ground. The foraging ants were either passively displaced posterior by head wind or pushed anterior by tail wind. At the same time the production of propulsive force was limited to the frictional properties of the substrate. (The maximum possible frictional force -being the product of normal force and the static frictional coefficient – was limited by the substrate used.) And indeed, ants that experienced head wind during their outbound run produced a high number of short steps. (see video, [Additional file 1]). Although challenged by constant backward displacement the animals maintained a highly coordinated gait pattern, which was qualitatively similar to locomotion on a rough substrate and resembled only temporarily the locomotion of water striders with front and hind legs resting and the middle legs "rowing" (e.g., [33]). When the ants were transferred to the parallel test channel they still experienced the wind, but – now successfully clinging to the rough ground – did not encounter slipping during running. They expected the nest close to the position where the ants foraging under wind-free conditions searched for their nest (Figure 5). The centers of the nest searches of the head wind ants (8.0 ± 1.90 m, n = 18) did not differ from the centers of search of the wind free ants (7.23 ± 1.22 m, n = 15) (p = 0.39; Mann-Whitney U-test; [34] Mann and Whitney, 1947). Compared to the zero control situation (outbound and inbound runs on rough ground, 9.1 ± 1.4 m), the wind free ants on slippery ground searched at an earlier stage of their inbound run (p = 0.0005; U-test). On the other hand, if the ants experienced tail wind during their outbound runs, they were only required to perform very few steps, what they also did in a highly coordinated manner (see video, [Additional file 2]). Their estimation of the nest entrance was far shorter (4.15 ± 2.46 m, n = 13) than that of the wind-free control ants (p = 0.0007; U-test). Under tail-wind conditions the ants underestimated the length of their outbound path. As each step on the micro-grit surface produced only a small and limited shear force, the number of steps required to reach the nest differed strongly with the experimental conditions. In consequence, the product of step number and stepping force is low in tail wind ants and high in head wind ants. This qualitative relationship is mirrored in the location of the centers of the search density distributions (Figure 4)

## Discussion

As our kinematic analyses of ants running on inclines show, the ants employ a very robust locomotor behavior that is almost not influenced by the inclination of the substrate. The major influence slope has on kinematic parameters is speed of locomotion. Faster running ants make longer steps and do so at a higher frequency. This robust relationship, which had already been shown for *Cataglyphis *species walking on level ground [19-21], remains present even if the ants are running on slopes. Once the effect of speed is removed, slope affects only temporal kinematic parameters. These effects are tiny and remain almost exclusively below the threshold set by the temporal resolution of the experimental setup.

### Footfall geometry and step length

The footfall geometry of the tarsi, both in the anterior and posterior extreme positions of the legs, differs mainly between the two species analyzed. This is a clear consequence of species-specific morphology. *Cataglyphis fortis *has comparatively longer legs than *Formica pratensis *has (Wehner and Sommer, in preparation). Hence *Cataglyphis *places its legs farther away from the body than *Formica *does. This difference in morphology allows the desert ant to make longer steps (see also: [35]). The values for step length on level ground obtained in the present study coincide well with the data presented by Zollikofer [19] for the same species. A similar effect can be found for individual body size as a covariant: larger animals make longer steps. The footfall geometry, however, is only partially affected by variations in body size. In conclusion, all geometric parameters are influenced by the morphology of the animals, and step length is additionally influenced by speed of locomotion. Contrary to the findings of Zollikofer [19], there is a slight influence of speed on some footfall parameters. This effect is mainly found in the middle legs: at higher speeds the middle tarsus is positioned closer to the body and hence can be placed anteriorly at AEP (anterior extreme position) and the stance is prolonged at PEP (posterior extreme position). However, the effect is small and may have been below the recording threshold of Zollikofer's experimental setup. When walking on an inclined surface, the vertical projection of the animal's center of mass will shift considerably. Nevertheless an according shift of the footfall geometry, the support base of the stepping tripod, could not be observed. In conclusion, the ants either adjusted the body height in order to keep the vertical projection of the COM within the supporting base, or they used their uphill legs to actively pull the body toward the surface. Force measurements on cockroaches running on inclined surfaces confirm that the uphill-positioned legs generate negative force to keep the body close to the surface and hence allow the animal to cling to the surface instead of "standing" safely [36]. The apparent shift in weight distribution on the legs (e.g., when the ant is walking uphill, the hind legs will carry the majority of the body load) did not result in a leg-wise change in step length either in our study animals or in the cockroaches. The positioning of the legs including step length was not influenced by slope.

In the Introduction we assumed that the working area of the legs and consequently the footfall geometry and/or the resulting step length may be influenced by locomotion on inclined surfaces. The ants could monitor this proposed effect by hair field sensors occurring at the leg joints. Although these joint-located hair sensors have been identified as graviceptive sensors in ants [25], our findings let us assume that measurement of the angular positions (i.e. the thorax-coxa joint) of the leg-plane will not reveal gravimetric information. Similarly to the results of Wittlinger and co-workers [20] these findings do not support the suggestions of Collett and co-workers [37], who argued that the angular working area of the leg-plane provides the animal with pedometer information.

In the present account we performed planar, i.e., two-dimensional kinematic analyses on the three-dimensional leg movements. Hence our data are strictly spoken, underdetermined. However, the femur-tibia and tibia-tarsus joints are parallel aligned hinged joints [38] causing femur, tibia, and tarsus to act within one plane, which is moved forward and backward by the movements of the thorax-coxa joint. Considering the conservative endeffector-positions of our results, i.e. the center of mass of the animal and the tarsus remaining at the same position, two types of movement remain possible: Tilting of the leg plane and variation in body height above ground. A parallel kinematic study of the sagittal plane [31,32] which showed no variation of body attitude and postural configuration excludes the latter possibility mentioned. Any hypothetical tilting of the leg plane could only be examined in a three-dimensional kinematic study. Nevertheless, based on the current state of the art, there is no indication of such an effect: Wittlinger and co-workers [20], who eliminated hair field sensors in both legs and body segments in foraging ants, were not able to causing any systematic errors in three-dimensional path-integration tasks. Considering the results the present study in conjunction with work of other groups the monitoring of inclination and step length via measurement of joint angle variations appears highly unlikely.

### Temporal coordination on inclines

The previously discussed geometric data obtained in our cinematographic studies show that for monitoring the inclination of their walking floor ants do not exploit the static positions of their legs. However, the cyclic movements of their legs and the linear acceleration of the whole body are subject to external forces such as gravity and hence might be speeded up or slowed down during locomotion on inclined surfaces. On level ground our results on temporal parameters such as swing and stance period, stepping frequency and phase relationships are – as the geometric parameters – in good accord with previous kinematic studies on ant locomotion [21]. With higher speeds cycle time is reduced (or frequency is increased), mainly by a shortening of the stance phase. In other words, the duty factor, a measure of static stability, decreases with speed. As ants run slower on inclined surfaces, especially on uphill paths, their duty factor is generally higher. We may speculate that if the ants choose to run slower on inclinations they do so for gaining higher stability than on level ground.

The conservative phase relationships between the six legs indicates that the gait, remains constant on all inclines examined: In both species the adjacent ipsilateral and the contralateral legs exhibit a phase shift of 0.5 which is characteristic for an alternating tripod gait. The ants maintain their gait, but show a small variability of leg coordination with speed. It is especially the front legs that touch down the later within the stepping cycle, the faster the ants run.

The two species differ, but only to a small extent, in the temporal parameters of locomotion. In wood ants the cycle period is shorter. This is likely due to the shorter and hence lighter legs. However, this effect disappears when frequency instead of cycle period is taken into account. These two fits are based on the very same data with one being the inverse of the other. But the model of cycle period has the by far higher log-likelihood-value and hence provides a better fit. From the data plotted in Figure 2 we conclude that frequency does not change linearly with speed, but its inverse – cycle period – does. Judging from the data plots the ants' maximum sustainable frequency lies in the range of about 25 Hz for both species. The species-specific shortening of the stance phase takes place at an even lower magnitude than the speed-specific reduction does, and hence can be considered irrelevant. Bigger animals have longer swing phases. This may be a consequence of the higher angular momentum of a longer and therefore heavier leg. Again, this factor is an order of magnitude smaller than the speed-influences. Slope has a tiny but statistically significant effect on cycle period and on both stance and swing phase. The statistical significance in our model refers to the quality of the fitted function compared to the measured data. In consequence, a functional non-influence can also be highly significant, for example, when the data form a constant line with the inclination zero. In this certain case, the values of the influence are tiny and close to zero. They almost exclusively do not supersede the threshold set by the experimental setup of 0.004s. When comparing cycle period between the steepest downhill (-60°) and the steepest uphill (60°) inclination the influence adds up to values between -0.006s and -0.008s. It was previously discussed that e.g. the efference copy of an altered motor output could serve as the desired correlate of step length and inclination [20]. In order to do so, we would expect that the influence is strong enough to differentiate (i) between inclined and level substrate and preferably (ii) between different absolute values of inclination. Although this mechanism seems appealing, our results do not necessarily support this hypothesis.

When running on an inclined surface, gravity has two effects on an animal's body: First, the body is pulled toward the surface on which the animal walks, but to a lower degree than if the animal were running on level ground; second, the animal's anterior movement is either impaired (or supported) by the substrate-parallel fraction of the externally acting gravitational force. If the ant could not compensate for this external influence it would, e.g., on uphill runs, show a prolonged stance phase, as active thrust is counteracted by downward acceleration or even a prolonged swing phase of the rather passively swinging legs. However, neither effect has been observed. The only effect that we observed lay in a certain variability of the relationship between step length and frequency in extreme situations of *Cataglyphis *locomotion: In steep uphill paths, when *Cataglyphis *is extremely slow, and on -30° downhill paths, when *Cataglyphis *runs rather fast, the step lengths at a given frequency do not coincide with the relationships of the other three conditions examined (Figure 2a). This effect may be due to energetic conversions: While on the -30° -slope the conversion from potential energy to kinetic energy is supported and hence longer steps are possible, the production of suficient potential energy during running on 60° -slopes impairs the ant from making long steps. However, this effect only occurs in *Cataglyphis*. It disappears at the other slopes examined. It might well be due to other factors such as the different sizes of the ants recorded during the different experimental sub-sets. Hence it will certainly not suffice for reliably monitoring surface inclination. Therefore, joint position sensors, even if their information were integrated over time, do not seem to qualify for inclinometric measurements employed by the ants in three-dimensional path integration.

### Ants do not employ special kinematics

Previous studies on insect kinematics revealed two basic types of locomotion: a highly controlled feed-back type of slow locomotion and a fast feed-forward locomotion that relies on 'mechanical intelligence' rather than on sensory control [39]. The fast runners examined were mainly cockroaches (*Blaberus discoidalis*; e.g. [40,41]), i.e., animals whose most elaborate navigational task is to escape bright light or approaching predators. On the other hand, the slow-walking stick insects employ highly elaborated control architectures leading them through their extremely complex habitats [42]. Running on inclined surfaces evoked an increase of the duty factor in climbing locusts [43], and an elongation of the stance phase on uphill paths in cockroaches and stick insects (stick insects: [44,45]; cockroaches: [46-48]). In potato beetles, *Leptinotarsa decemlineata*, running uphill showed a small statistically insignificant decrease of the stance phase as well as an inclination-dependent variation of the footfall positions [49]. While the the fast-running insects maintain constant locomotion patterns in virtually all environments and cope with disturbances by purely mechanic means, the latter insects employ highly variably kinematics in order to keep themselves on track. Desert ants, however, have to meet contradictory demands: their habitat does not provide shelters to heat and predators, so that these animals have to employ both the high speeds of the cockroach [41] and a high sensorial control over their movements as exhibited by stick insects [42], as only precise path integration and fast running will allow them to get back to their safe nest in time. Now, what is the type of runner that the desert ants' locomotor system resembles most? Or would there even be an intermediate type allowing for high sensorial feedback at high running speeds? Or would there be different running styles in the far-ranging desert ants in their open environment and in the landmark-following wood ants in their cluttered environment (as is the case in arboreal and terrestrial lizards: [50])? Our results lead to a clear-cut answer: desert ants as well as wood ants employ the cockroach-type of locomotion. Hence, this style of fast feed-forward locomotion still allows for sensorial records of each single step, its corresponding length and the inclination of the substrate, even though the pattern of leg movements does not vary. The by far most prominent effect the inclined surface has on the ant's walking parameters are the different speeds the animals choose to take. The speeds recorded for *C. fortis *in the present account differ somewhat from the data obtained by Wohlgemuth and co-workers [24], who studied ants foraging within a linear array of hills. In both studies *C. fortis *ants ran at about 0.2 m/s (with maxima up to 0.4 m/s) and reduced their walking speed on uphill slopes, but in the latter study the values are somewhat lower (0.05 and 0.1 m/s respectively, at ≈60°). On downhill slopes, in either study the speed did not depend on the degree of the slope. In the present account the ants ran at 0.23 m/s and hence twice as fast as the Wohlgemuth ants. The setup of Wohlgemuth and co-workers [24] consisted of a series of hills which might have triggered the ants to run at lower speeds in order to save energy. The tendency to run slow on uphill slopes and to keep a fair speed of travel on downhill slopes is apparent in both studies. Energy recovery, i.e., the conversion of potential energy to kinetic energy, would allow the ants to save energy on downhill slopes. While potential energy recovery is well known in bigger animals (e.g. turkeys: [51]), ants do not seem to use a substantial amount of the potential energy for propulsive means [52].

### Locomotion on slippery ground

The last hypothesis we had proposed for slope detection assured that the walking ants somehow measured forces acting on their legs. Indeed maintaining a constant movement pattern and hence compensating external influences requires variability in force production. When in our experiments the ants were running on a slippery surface, the production of shear force was limited by the low frictional coefficient of the substrate. Since the maximum static frictional force (a product of normal force and static frictional coefficient) was easily superseded within this setup, each stepping attempt of an ant resulted in the production of a constant force output. In addition to that, external (in our case wind-load) forces pushed the ant forward or backward and hence varied effective step length. The results of this particular pilot study show that ants, when running with strong head winds and hence producing many short steps, integrate the very same nest-feeder distance as the wind-free ants do. When foraging under strong tail-wind conditions, the ants will produce only very few but rather long steps and underestimate the nest-feeder distance considerably. Both head-wind and wind-free ants ran for shorter distances than the zero control ants did. In contrast to the latter group the first two groups of ants were confronted with different floor textures and colors on the channel ground during outbound and inbound situations. As Seidl and Wehner [53] had already shown, inbound ants have an expectation of the visual and tactile ground properties. Hence, in the present study the ants' behavior should have been influenced by the mismatch between the slippery white outbound floor and the rough light-brown inbound floor. Due to technical reasons it was not possible to record the ants' entire outbound runs, which would have allowed us to estimate the actually performed number of steps and the corresponding step lengths. However, our results show at least qualitatively that horizontally acting forces could be the desired correlate for step length, and that if the relationship between stepping force and step length is disturbed, the ants miscalculate distances. Force sensors involved in graviception and kinesthetic orientation have been described to occur in arthropod legs (insects: [26]; spiders: [54]), and muscular strain modulation on inclines has been reported in vertebrates [55]. Hence, we finally suggest that such sensors – e.g. campaniform sensilla or muscular strain sensors – should be the main target of future experiments on the question of how navigating desert ants monitor step length and record surface inclination.

## Methods

### Kinematic analysis

**Figure 6:**
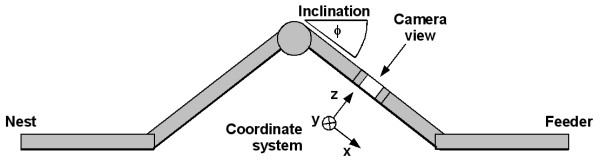
Schematic drawing of the setup used in experiment 1 (side view). While foraging from the nest to the feeder the ants passed a narrowed section of the channel, within which they were recorded from above (x-y plane, see arrow). The inclination of this section could be altered from level ground (*φ *= 0°) to almost vertical (*φ *= 90°).

High-speed video recordings were performed in August 2004 at our field station near Maharès in southern Tunisia (nest coordinates: N 34° 31.745', E 10° 32.333') on desert ants, *Cataglyphis fortis*, and in October 2003 at the University of Jena (Germany) on wood ants, *Formica pratensis *(Retzius 1783), the so called Black-Backed Meadow ant. We used active foragers of established colonies, which were linked to a channel array. The animals were trained to forage to a feeder at the end of a U-shaped aluminum channel (profile dimensions: 7 by 7 cm). During foraging, outbound ants were recorded from a dorsal perspective when they passed a narrowed part of the channel. In addition, the inclination of this part of the channel could be varied, so that ants foraging on differently inclined floors could be recorded (Figure 6).

### Data acquisition and analysis

**Figure 7:**
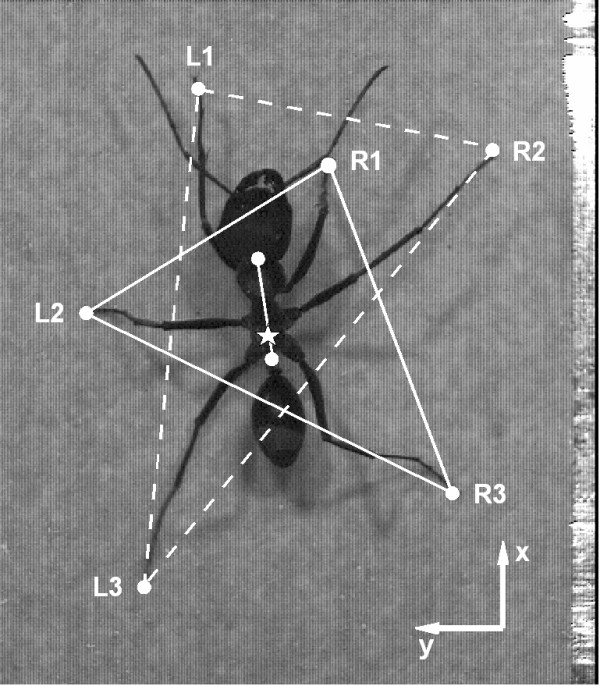
Dorsal view of *C. fortis *foraging in the channel arrangement. For kinematic analysis the head-thorax joint and the petiolus were tracked as well as the contact point of the tarsi of all six legs with the ground (L1 through R3 denoting left and right legs, and front to hind legs, respectively).

The ants' movements were recorded with a high speed camera (Redlake PCI 2000 S) at 250 frames per second. Approximately 100–150 sequences of freely foraging outbound ants were taken for each inclination. The nests used in this experiment contained about 70 to 100 active foragers per day. For kinematic analysis only sequences with straight and uninterrupted runs were chosen. The sequences were tracked in the laboratory of Reinhard Blickhan (Friedrich Schiller University Jena, Germany) using WinAnalyze (Mikromak) tracking software. For the analysis of the stepping parameters the head-thorax joint and the petiolus were tracked, and so were the contact points of the tarsi of all six legs with the ground (Figure 7). Prior to each series of recordings the setup was calibrated by recording small plastic blocks (Lego systems) that filled almost the entire field of view. During tracking, data were calibrated by a linear measure of the calibration body. Raw data were exported and analyzed using custom written programs implemented in Matlab (The Math Works Inc.). The center of mass (COM) of each animal was determined as 1/10 of the vector from the petiolus to the head-thorax joint. This caused the origin of the coordinate system to rest between the coxae of the middle and the hind leg pair, described as the animal's COM [21]. The the x-axis of the coordinate system was aligned to the body axis. Additionally, for each sequence analyzed the width of the ant's head was determined from the video sequences. This measure is linearly correlated with body size [1]. We refrained from calculating values for a standardized body model as (i) the scaling factors differ slightly between the front, middle, and hind legs and also between the two species. Furthermore, as we (ii) took freely foraging animals, we were not able to take measurements on, e.g., alitrunk length or leg length. However, in the dorsal views of our recordings absolute head width can be determined easily. Hence in our statistic model head width serves as a covariant for individual body size.

### Modeling stepping parameters

Stepping patterns are influenced by speed, but may also vary between differently sized animals and between animals of different species. In order to separate these effects from a possible effect that slope could have on stepping patterns, we analyzed each examined parameter (see Tables 1 and 2, first column) with the four input variables speed, head width, species, and slope. As the data set is unbalanced, we performed fits with reduced maximum likelihood-methods (LME-function, NLME-package; [56]) implemented in the statistical software package R [57] in the following form:

*Parameter *= α * *Speed *+ β * *Species *+ γ * *Headwidth *+ δ * *Inclination *+ *ε*

The input-variable 'Species' is a factor and not a value, and hence the animals are ordered alphabetically ('C' for *Cataglyphis fortis *and 'F' for *Formica pratensis*). A negative *β *indicates that the fitted parameter is smaller in *F. pratensis *than in *C. fortis*. All other variables used throughout this study are numeric. Goodness of fits can be compared with the log-likelihood given for each fit (Tables 1 and 2; last column), with a higher value indicating a better fit (assuming identical degrees of freedom, which is the case in our analyses). Steps within one single run were nested, i.e. not treated independently. Each single input variable fitted to a certain parameter has its own level of significance. In the results we only mention fitted parameters with levels of significance p < 0.1. For most of the parameters, data of the front, middle, and hind legs were used irrespective of whether they referred to the left or right side of the body. Footfall geometry was analyzed on legs that belonged to one tripod of the typical gait of fast running insects (and also of *Cataglyhis *ants, see [21]). Hence we chose the right front leg (R1), the left middle leg (L2), and the right hind leg (R3), which together formed one of the ant's locomotive triangles. For the analysis of phase relationships, we determined the phase shift of all legs with respect to the left middle leg (L2).

#### Behavioral Experiment

In the second type of experiment performed in this study *Cataglyphis fortis *ants were trained to forage to a feeder 9 m from the nest entrance. During the whole foraging excursion the animals were enclosed in a linear flat channel (material and dimensions: see above), in which the ground was covered with very fine abrasive paper (grit size: 100 nm; Manufacturer: Sia Microtec AG, CH-Frauenfeld) that minimized frictional forces between tarsi and ground [58]. This analogue to a larger animal walking on ice causes the ants to walk with highly increased slip rates, especially during phases of acceleration and deceleration. In order to amplify the slip rate even further, the foraging runs took place under different wind conditions (head wind and tail wind; both and wind speeds of 3–4 m/s; at speeds above 5 m/s *Cataglyphis *will usually not initiate foraging runs, personal observations) resulting in an extreme increase or decrease of the number of steps required by the animal in order to cover the same 9-m nest-feeder distance. The wind was constantly monitored via a vane; both the training- and the test channel were aligned parallel to the wind direction. The interior of the channel (walls: spray-painted with gray varnish, ground: 100-nm grit abrasive paper) did not provide the ants with any visual flow field cues that could have provided a feedback on running efficiency. When an ant had reached the feeder and had taken a food item (usually a melon flavored biscuit crumb), it was transferred to a parallel test channel that was twice as long as the training channel and contained a rough ground surface. Once released, each ant directly ran off its home vector, i.e., its representation of the distance from the feeder to the nest entrance. When the home vector had reached zero-state, the ant started a symmetric search centered on the expected nest entrance. Six turns within the ant's linear search pattern were recorded, and search density distributions were computed for each experimental set (for details see [53]).

## Competing interests

The authors declare that they have no competing interests.

## Authors' contributions

TS and RW designed the experiment, TS conducted the experiment and analyzed the data, TS and RW wrote the manuscript.

## Supplementary Material

Additional file 1 Outbound *C. fortis *experiencing head-wind on a slippery substrate. Click here for file

Additional file 2 Outbound *C. fortis *experiencing hind-wind on a slippery substrate. Click here for file
